# A case of pazopanib-induced acute kidney injury, reversible hair depigmentation and radiation recall dermatitis

**DOI:** 10.1080/0886022X.2023.2213778

**Published:** 2023-06-02

**Authors:** Jun Li, Zhiying Li, Tao Su

**Affiliations:** a Department of Geriatric Medicine, Peking University First Hospital, Beijing, China; b Renal Division, Department of Medicine, Peking University First Hospital, Beijing, China; c Institute of Nephrology, Peking University, Beijing, China

Dear Editor,

Pazopanib is a multitargeted tyrosine kinase inhibitor (TKI) that acts against vascular endothelial growth factor (VEGF), platelet-derived growth factor, fibroblast growth receptor, and c-Kit [1]. Before the guidelines were updated to recommend immune checkpoint inhibitors, pazopanib was initially approved as first-line therapy for advanced or metastatic renal cell carcinoma (mRCC) by the Food and Drug Administration (FDA) and recommended by the National Medical Products Administration of China [2]. However, the clinical efficacy of pazopanib is limited due to its association with adverse events (AEs), including kidney involvement [3]. Nevertheless, real-world evidence has shown that the presence of AEs is an independent predictive marker of better progress-free survival in mRCC [4]. Here, we describe a typical case of pazopanib-induced AEs, including acute kidney injury (AKI), hair depigmentation, and radiation recall dermatitis (RRD).

A 51-year-old Chinese man was admitted to Peking University First Hospital due to kidney dysfunction and edema. The patient had a medical history that included 6 years of hypertension and was therefore receiving treatment with nifedipine sustained-release tablets. The patient had been diagnosed with mRCC 3 years prior and had undergone a nephrectomy of the right kidney. During follow-up, metastases to the lung and ilium were detected one year later. Thus, treatment with sunitinib (50 mg/daily) was initiated with periodic radiation therapy; however, treatment was stopped because sunitinib failed to block metastases during the subsequent year. The patient presented with sunitinib-induced AEs, and his serum creatinine level gradually increased from 123 μmol/L (initiation of sunitinib) to 203 μmol/L, along with hypertension, 2+ proteinuria, and trace glycosuria. The left pelvic metastasis was resected, and artificial hemipelvic prosthesis replacement was performed. Pazopanib was recommended as a rescue treatment six months after sunitinib withdrawal. Unfortunately, the patient developed hair depigmentation one week after pazopanib treatment (800 mg/daily) (Figure 1(A,B)). Laboratory examination showed that his serum creatinine level had increased to 635.0 μmol/L 4 weeks later (Figure S1).

**Figure 1.**
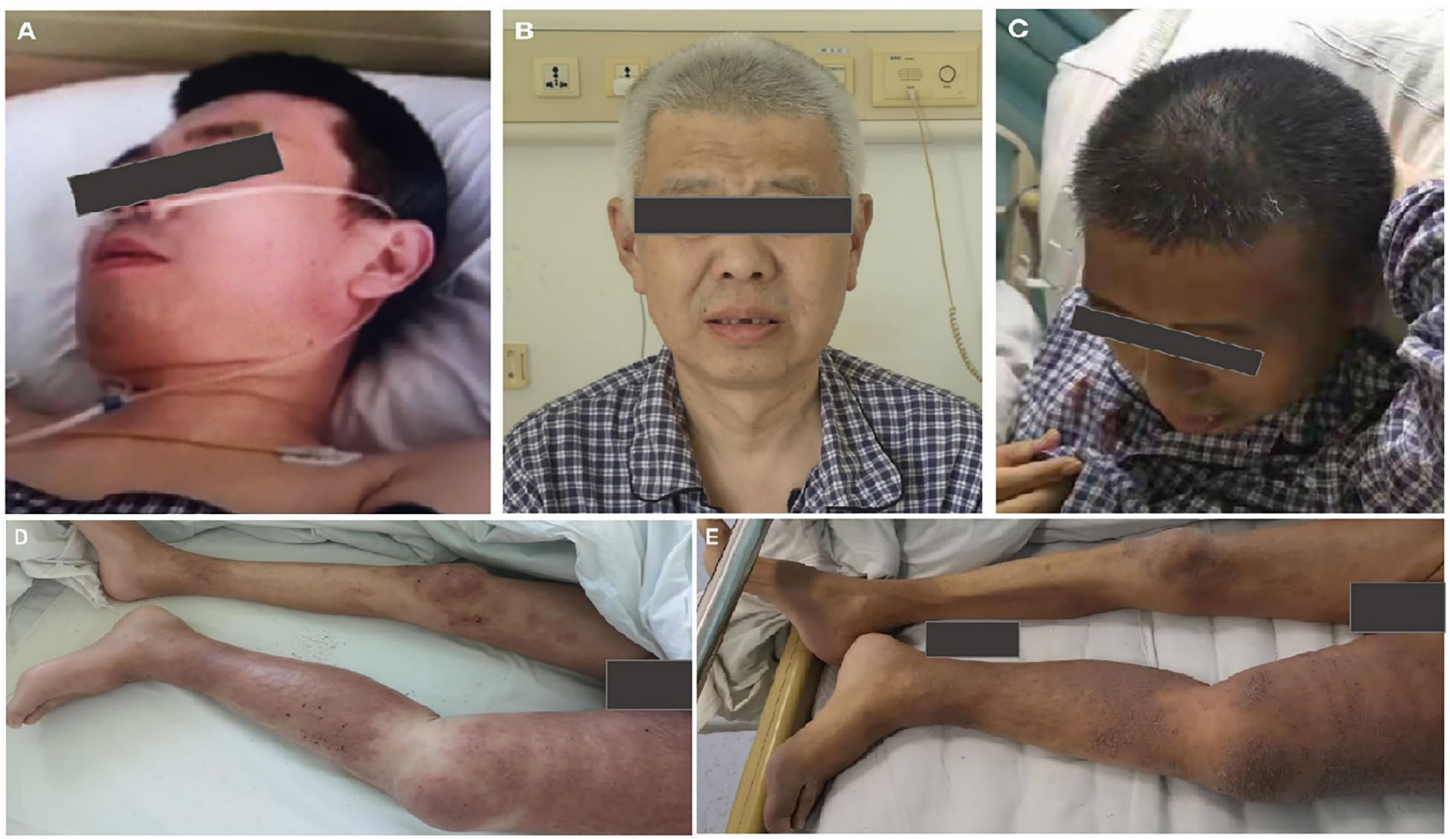
The hair depigmentation (A–C) and the radiation recall dermatitis (D,E) before and after taking pazopanib.

On admission, the patient was white-haired and had asymmetrical edema of both lower extremities, with edematous erythema distributed mainly on the left lower extremity with scratches (Figure 1(D,E)). Laboratory tests revealed anemia (hemoglobin, 82 g/L) and eosinophilia (eosinophil count, 1.8 × 10^9^/L) with normal white blood cell and platelet counts. Urinalysis revealed massive proteinuria (10.98 g per day) and 2+ renal glycosuria without hematuria. Exfoliated tubular epithelial cells were occasionally observed in the urine sediment. Urinary small-molecule proteins, albumin, and large-molecule proteins accounted for 10.9%, 51.1%, and 38.0% of total proteinuria, respectively. Biochemical tests revealed that serum albumin was 30.2 g/L; lactate dehydrogenase (LDH) was 243 IU/ml (100–240 IU/L); and the levels of glucose, phosphate and chloride were in the normal range. No monoclonal immunoglobulins were detected in serum or urine samples. No schistocytes, disorders of ADAMTs13 activity or complement factor H activity were observed. Autoantibodies, including anti-phospholipase A2 receptor, anti-glomerular basement membrane, anti-neutrophil cytoplasmic, antinuclear, antiphospholipid, anti-complement factor H antibodies, and rheumatoid factor, were negative. Ultrasound and magnetic resonance imaging revealed prosthetic dislocation of the left femoral head, left hip joint effusion, and swelling of the surrounding soft tissue. The solitary left kidney was 10.5 cm in length and 4.8 cm in width. No cancer-related venous thromboses or abdominal metastases were detected.

Based on a literature review, pazopanib was identified as a trigger for the emergence of AKI, superimposed on chronic kidney disease (proteinuria, renal glycosuria) and hair depigmentation. Given that the skin lesions were localized around the region of irradiation in the lower extremities, particularly on the left side of the body around the left humerus, and manifested as edematous erythema with eosinophilia, RDD was diagnosed. RRD can appear following treatment with pazopanib. We speculated that bilateral asymmetric edema of the lower extremities was caused by prosthesis dislocation of the left femoral head, leading to left hip joint effusion and lymphatic reflux disorder. The patient refused to take prednisone due to concerns regarding prednisone-related adverse effects and was given supportive measures, including diuretics, phosphate binders, and red cell transfusion. During his hospital stay, we observed spontaneous remission of depigmented hair, edematous erythema (Figure 1(C)), renal glycosuria, and eosinophilia, with the exception of the restoration of kidney function. The patient underwent hemodialysis.

Pazopanib, a selective multitargeted receptor TKI that blocks tumor growth and inhibits angiogenesis, has been widely used in patients with mRCC. Pazopanib-induced hair and skin color changes have been reported in 8.4%-38% of patients [5] and are caused by the inhibition of c-Kit, which exerts a powerful influence on melanocyte proliferation, migration, and differentiation in the developing hair and skin follicles [6].

Despite its extensive use, pazopanib-induced RRD and kidney dysfunction have seldom been reported. Reports of such cases, published from January 2009 to December 2022 in PubMed/MEDLINE, are summarized in Table S1. A rash occurs in approximately 5% of patients taking pazopanib and is related to a compromised immune system, abnormal immune cell function, and the release of many inflammatory mediators. RRD can be triggered by the administration of a broad range of systemic drugs, including pazopanib, after radiotherapy; however, the underlying mechanism remains unknown. In the cases highlighted in Table S1, prednisolone was effective against the rapid resolution and recurrence of rashes during pazopanib treatment. Proteinuria is the most commonly reported kidney adverse event linked to the TKIs pazopanib and sunitinib [7,8]. In a previous study, biopsied patients shared typical pathological features, such as diffuse glomerular microangiopathy, segmental glomerular capillary microaneurysms, and hyalinosis, with different extents of podocyte damage [9], while preglomerular arteriole microangiopathy and microthrombosis were rarely observed, regardless of whether they developed AKI. Abnormal crosstalk between endothelial cells and podocytes mediates TKI-induced proteinuria and kidney injury *via* blockage of VEGF receptors and their signaling. In the case described here, the patient’s history of sunitinib-induced AEs, including hypertension, proteinuria, renal glycosuria, and progressive chronic kidney disease, was suggestive of TMA. However, carrying out a biopsy of the solitary kidney to obtain a definite diagnosis was unfortunately not a suitable option for this patient, who had a poor mRCC outcome. It should be noted that the presence of a solitary kidney, preceding chronic kidney disease related to hypertension and sunitinib, as well as uncontrolled malignancy, all contribute to poor kidney outcomes.

In conclusion, this study reports an example of pazopanib-induced AEs showing massive proteinuria with AKI, reversible hair depigmentation, and RRD. These findings highlight the need for further attention from oncologists and nephrologists when making therapeutic decisions.

## Supplementary Material

Supplemental Material

Supplemental Material
