# Supplementary figures and images for: A case of pazopanib-induced acute kidney injury, reversible hair depigmentation and radiation recall dermatitis
Source: Ren Fail. 2023 Jun 2;45(1):2213778. doi: 10.1080/0886022X.2023.2213778 (PMC10240991; doi:10.1080/0886022X.2023.2213778)

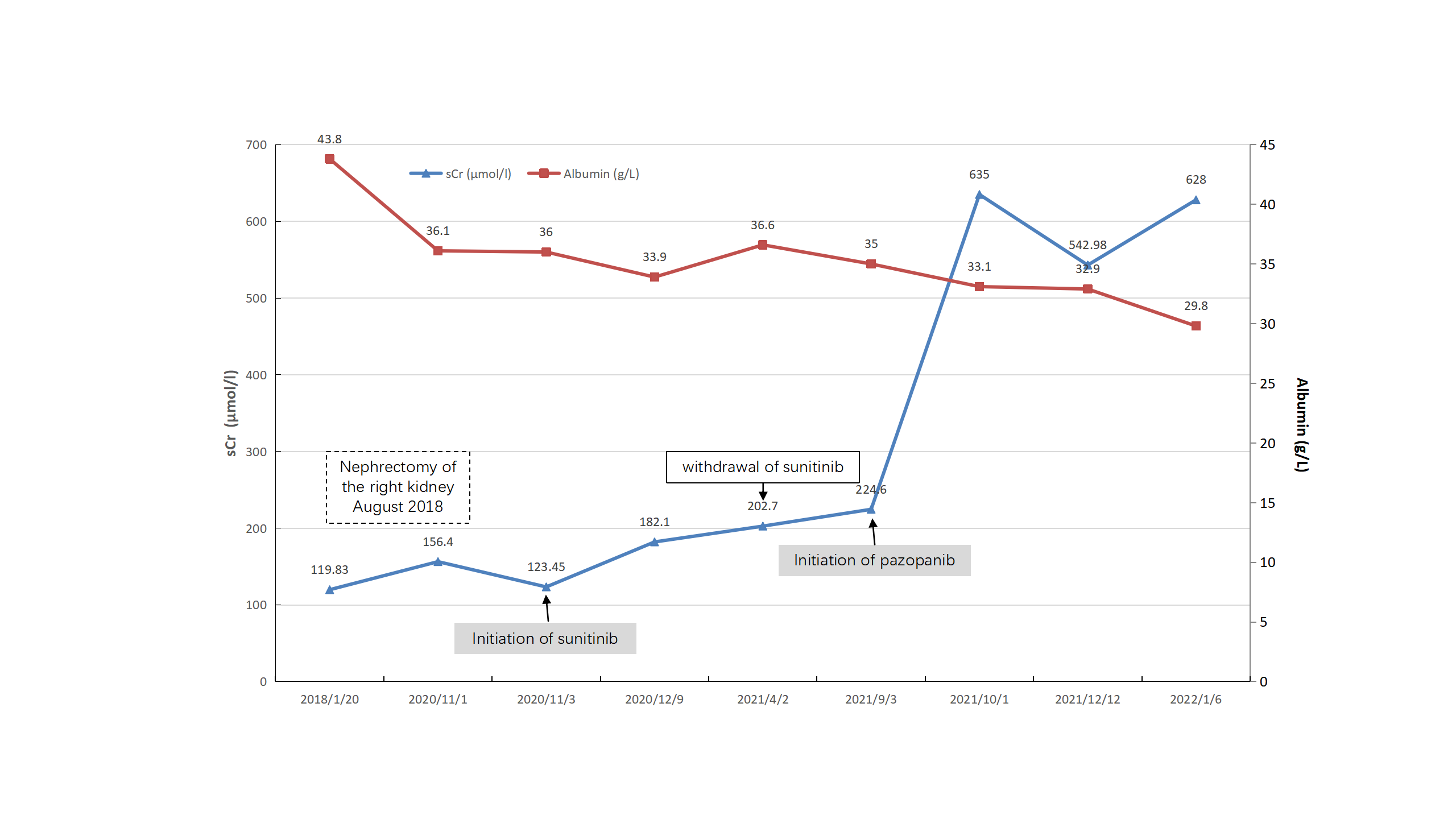

Supplement: Supplemental Material [file IRNF_A_2213778_SM5325.tif]
