# Supplementary material for: A case of pazopanib-induced acute kidney injury, reversible hair depigmentation and radiation recall dermatitis
Source: Ren Fail. 2023 Jun 2;45(1):2213778. doi: 10.1080/0886022X.2023.2213778 (PMC10240991; doi:10.1080/0886022X.2023.2213778)
Supplement: Supplemental Material [file IRNF_A_2213778_SM5323.pdf]

Table S1. Published cases of pazopanib-induced kidney dysfunction and radiation recall dermatitis

Study	Sex	Age range	Cases	Diagnosis	Clinical presentation	Pathology
Bible KC [1]	F/M	23-79	3/37	Advanced differentiated thyroid cancers	Raised creatinine concentration	---
Kalla S[2]	M	50-55	1	mRCC	AKI, nephrotic range proteinuria, hypertension, and fluid overload	TMA
Syed U[3]	M	75-80	1	mRCC	AKI, hyperbilirubinemia and thrombocytopenia	---
Buti S [4]	N.D	27-86	6/48	Non-clear cell renal carcinoma	Renal failure	---
Pfister F [5]	M	75-80	1	RCC	Proteinuria, renal dysfunction	TMA
Sleijfer S [6]	N.D	N.D	37/140	Soft tissue sarcoma	Kidney AE Grade 1-3*	
Pal SK [7]	N.D	N.D	16/28	mRCC	1 with AKI; 15 with elevation of creatinine	
Azad A [8]	M	60-65	1	mRCC	RRD	---

Information Classification: General

Haraldsdottir S [9]	M	60-65	1	Metastatic, non–small-cell lung adenocarcinoma of the bone and thoracic lymph nodes	RRD		eccrine squamous syringometaplasia, epidermal dysmaturation, and radiation-associated changes

Note: Our search included a combination of keywords such as "pazopanib", "skin", "dermis", "renal" and "kidney". Our search was limited to English articles including case reports, original articles, and review articles. Reported skin and kidney presentations which were irrelevant to pazopanib or did not contain detailed information were excluded. F, Female; M, Male; N.D, no detail data; AKI, Acute kidney injury; RCC, Renal cell carcinoma; mRCC, Metastatic or advanced renal cell carcinoma; TMA, thrombotic microangiopathy. RRD, radiation recall dermatitis.

* graded according to National Cancer Institute Common Terminology Criteria of Adverse Events version 3.0.

REFERENCES

1. Bible KC, Suman VJ, Molina JR, Smallridge RC, Maples WJ, Menefee ME, Rubin J, Sideras K, Morris JR, McIver B *et al*: **Efficacy of pazopanib in progressive, radioiodine-refractory, metastatic differentiated thyroid cancers: results of a phase 2 consortium study**. *LANCET ONCOL* 2010, **11**(10):962-972.
2. Kalla S, Ellis RJ, Campbell SB, Doucet B, Isbel N, Tie B, Jegatheesan D: **Thrombotic Microangiopathy Associated with Pazopanib in a Kidney Transplant Recipient**. *J Kidney Cancer VHL* 2021, **8**(1):25-31.
3. Syed U, Wahlberg KJ, Douce DR, Sprague JR: **Thrombotic Thrombocytopenic Purpura Associated with Pazopanib**. *Case Rep Hematol* 2018, **2018**:4327904.
4. Buti S, Bersanelli M, Massari F, De Giorgi U, Caffo O, Aurilio G, Basso U, Carteni G, Caserta C, Galli L *et al*: **First-line pazopanib in patients with advanced non-clear cell renal carcinoma: An Italian case series**. *World J Clin Oncol* 2021, **12**(11):1037-1046.
5. Pfister F, Amann K, Daniel C, Klewer M, Buttner A, Buttner-Herold M: **Characteristic morphological changes in anti-VEGF therapy-**

Information Classification: General

induced glomerular microangiopathy. *HISTOPATHOLOGY* 2018, **73**(6):990-1001.

6. Sleijfer S, Ray-Coquard I, Papai Z, Le Cesne A, Scurr M, Schoffski P, Collin F, Pandite L, Marreaud S, De Brauwer A *et al*: **Pazopanib, a multikinase angiogenesis inhibitor, in patients with relapsed or refractory advanced soft tissue sarcoma: a phase II study from the European organisation for research and treatment of cancer-soft tissue and bone sarcoma group (EORTC study 62043)**. *J CLIN ONCOL* 2009, **27**(19):3126-3132.
7. Pal SK, Hossain DM, Zhang Q, Frankel PH, Jones JO, Carmichael C, Ruel C, Lau C, Kortylewski M: **Pazopanib as third line therapy for metastatic renal cell carcinoma: clinical efficacy and temporal analysis of cytokine profile**. *J Urol* 2015, **193**(4):1114-1121.
8. Azad A, Maddison C, Stewart J: **Radiation recall dermatitis induced by pazopanib**. *Onkologie* 2013, **36**(11):674-676.
9. Haraldsdottir S, Bertino E, Haglund K, Kaffenberger B, Shah MH: **Radiation Recall Dermatitis With Concomitant Dabrafenib and Pazopanib Therapy**. *JAMA DERMATOL* 2016, **152**(5):587-589.

Information Classification: General
